# Anticancer and Immunomodulatory Benefits of Taro (*Colocasia esculenta*) Corms, an Underexploited Tuber Crop

**DOI:** 10.3390/ijms22010265

**Published:** 2020-12-29

**Authors:** Patrícia Ribeiro Pereira, Érika Bertozzi de Aquino Mattos, Anna Carolina Nitzsche Teixeira Fernandes Corrêa, Mauricio Afonso Vericimo, Vania Margaret Flosi Paschoalin

**Affiliations:** 1 Biochemistry Department, Chemistry Institute, Federal University of Rio de Janeiro (UFRJ), Av. Athos da Silveira Ramos, 149, sala. 545, Cidade Universitária, Rio de Janeiro (RJ) CEP 21941-909, Brazil; patriciarp@iq.ufrj.br (P.R.P.); erika_bertozzi@hotmail.com (É.B.d.A.M.); annac.correa@hotmail.com (A.C.N.T.F.C.); 2 Biology Institute, Fluminense Federal University (UFF), R. Alexandre Moura, No. 8, Bloco M, sala. 505, Gragoatá, Niterói (RJ) CEP 24210-200, Brazil; mavericimo@gmail.com

**Keywords:** health-promoting compounds, resistant starch, metabolism modulation, COX-2 down-regulation, taro dietary intervention

## Abstract

Taro corms contain valuable bioactive molecules effective against cancer and cancer-related risk factors, such as carcinogens and biological agents, several pathophysiological conditions, including oxidative stress and inflammation, while controlling metabolic dysfunctions and boosting the immunological response. Such broad effects are achieved by the taro health-influencing compounds displaying antitumoral, antimutagenic, immunomodulatory, anti-inflammatory, antioxidant, anti-hyperglycemic, and anti-hyperlipidemic activities. Taro bioactivities are attributed to the combination of tarin, taro-4-I polysaccharide, taro polysaccharides 1 and 2 (TPS-1 and TPS-2), A-1/B-2 α-amylase inhibitors, monogalactosyldiacylglycerols (MGDGs), digalactosyldiacylglycerols (DGDGs), polyphenols, and nonphenolic antioxidants. Most of these compounds have been purified and successfully challenged in vitro and in vivo, proving their involvement in the aforementioned activities. Although these health-promoting effects have been recognized since ancient times, as well as other valuable features of taro for food profit, such as hypo-allergenicity, gluten-free, and carbohydrates with medium-glycemic index, taro crop remains underexploited. The popularization of taro intake should be considered a dietary intervention strategy to be applied to improve the overall health status of the organism and as supportive therapy to manage tumorigenesis.

## 1. Introduction

### 1.1. Underutilized and Neglected Crops Worldwide

Crops that have been neglected over the years are currently being revalued based on modern technologies used to extract, identify, estimate, and assay a great number of compounds displaying claimed pharmacological effects. The study of the composition of such food matrices has stimulated the recognition and reevaluation of so-called “orphan” crops, reaffirming the knowledge that traditional communities have practiced since ancient times by considering the vital role of those crops not only in supporting diets but also in promoting the health and treating these populations. In most cases, neglected or underutilized species have been substituted by those cultures in huge demand, although sometimes, those crops are poorer not only in nutritional aspects but mainly in bioactive compounds [1].

### 1.2. Taro Consumption, Cultivation, and Nutritional Importance

Even though taro corm (or taro) is a rich source of health-promoting compounds, this crop, as well as tubercle consumption worldwide, is highly neglected probably because it is mainly associated with subsistence agriculture [2,3,4]. Moreover, due to poorness, unsustainable farming practices, and climate change, taro crops face many challenges in several underdeveloped countries, such as African Sub-Saharan nations and other countries in Central and South America [5]. In general, taro crops, as several subsistence crops, are cultivated in small farms, with low capital endowment, far from urban centers and with no access to capital markets, and low-off farm income [1]. The food processing sector can overcome these constraints and enhance taro crop availability and acceptance by urban populations by replacing corn and wheat in processed foods, enhancing raw product commercialization. In addition, this may also lead to attention regarding taro crops as a rich source of remarkable and unique compounds, whose pharmacological activities have been demonstrated both in in vitro and in animal models.

International research centers mainly dedicated to taro studies are still scarce, although they would be helpful to overcome many challenges that have remained unsolved for over ten years. Financial and scientific investments would aid in improving cultivation conditions, creating and maintaining germplasm collections of diverse regions, improving conservation methods, increasing food security, and enhancing the benefits of taro consumption. These efforts would increase the research field and shared information between countries, which might expand taro cultivation, sales, and consumption worldwide, especially in developing countries [6].

**Figure 1:**
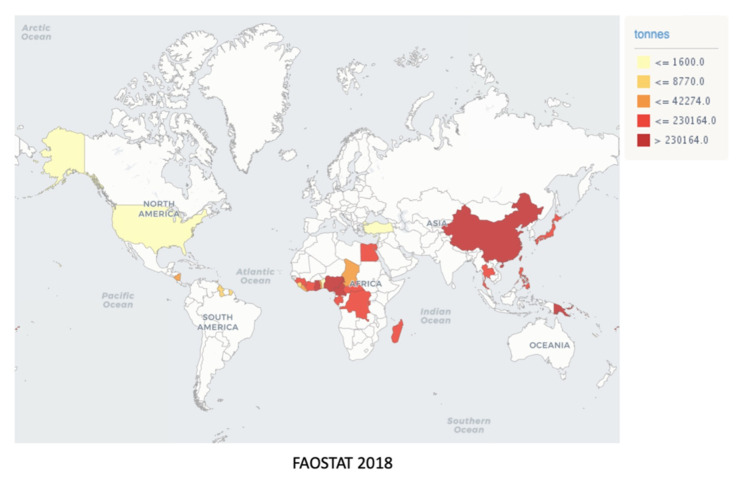
Global distribution of taro production reproduced from FAOSTAT (http://www.fao.org/faostat/en/#data/QC). Quantitative taro production per country in 2018, repreScheme 230. tons followed by the USA, Canada, and Cyprus with production lower than 1600 tons. Uncolored countries represent production areas under 1000 ha.

The most significant taro producers are the West African countries, i.e., Nigeria, Cameroon, and Ghana, followed by China, which contribute respectively 6.7 and 3.9 million tons of taro, corresponding to 83.6% of the worldwide taro production [7,8]. Other nations, such as the USA, Canada, Japan, Turkey, and Central and South American countries, produce about 2 million tons of taro. Brazil has not yet been internationally recognized as a taro producer country, since less than 1000 ha are planted and dispersed, which is probably due to the vast Brazilian territory, where relevant producers are spread throughout the Mid-South region (Figure 1) [9,10]. However, Southeast Brazil boasts a germplasm bank, named INCAPER, which is used to collect and conserve taro cultivars, maintaining the diversity and characteristics of the Brazilian varieties, which include T37 (Macaquinho), T38 (Chinês), T39 (Japonês), T40 (Chinês Regional), T41 (Cem em Um), T42 (São Bento), and T43 (Branco) [11]. The neglected and underutilized *status* of taro crops is noted by comparison to other tubercles, such as potatoes, which are widely consumed worldwide, although displaying superior nutritive importance. For example, in 2018, approximately 12.6 million tons of taro per annum were produced worldwide against 64.7 million tons of potato (*Solanum tuberosum* L.) (Figure 1) [7]. Taro is a healthy alternative of carbohydrate source, as the cooking process does not interfere with their nutritional composition, causing only minimal modifications in nutrient contents, according to Food Data Central from the United States Department Agriculture (USDA) (https://fdc.nal.usda.gov/) [12]. The proximate composition of crude, cooked, and baked taro is quite similar regarding vitamins and minerals, except for niacin and calcium levels, as well as protein and total fat amounts, which were lowered by thermal processing (Table 1). 

Additionally, taro is a rich source of antioxidants, mainly phenolic compounds, both regarding diversity and quantity, distributed in the edible portion of taro. In addition to antioxidants, taro phytochemicals display immunomodulatory, antioxidant, antitumoral, antimetastatic, antimutagenic, anti-hyperglycemic, and anti-hypercholesterolemic bioactivities. Moreover, taro is a potential alternative staple source, with a lower glycemic index than potato, and its consumption may decrease the incidence and prevalence of several diseases, including certain types of cancers [13,15,16,17]. 

Despite being considered an orphan crop, taro is a sacred food in some cultures, such as in Hawaii, Melanesia, and Micronesia, where it is known as a Gift of Ancient Gods. In these places, taro is consumed daily and included in several special occasions and rituals due to its symbolic importance [10]. Taro is formulated according to the cultural traditions of each local population. For example, taro stems, petiole, corms, and leaves can be consumed as a common practice in Hawaii. However, taro corms are conventionally considered the edible portion of this plant, and they are consumed worldwide [2]. Some cultivars can exhibit high calcium oxalate contents, which is considered an antinutritional factor that confers an acrid taste to the tubercles, causes skin irritation, and can decrease calcium absorption [18]. For this reason, taro should be preferentially consumed after cooking in order to avoid these undesired effects.

In Hawaii, taro is cooked and smashed with a little water to prepare a starchy paste, which may be consumed immediately (fresh poi) or after 2–3 days of fermentation producing a sour taste paste (sour poi), which is a typical Hawaiian dish [19]. Achu, another ancient taro paste, preferentially prepared by women, is mostly consumed in Africa. Taro and bananas are boiled together, peeled, and pounded to form a smooth and homogeneous starchy paste. Then, typical sauces are mixed in, such as yellow sauce (achu soup), jaune sauce, black sauce (black soup), and pepper sauce [20]. 

In other parts of the world, especially Brazil, taro can be served fried or steamed, prepared as a soup, or mashed. The corms are also marketed in a variety of commercial products such as flour, chips, fermented alcoholic beverage, ice bar, ice cream and canned taro, among others [21,22]. These taro derivatives are not globally available, as taro crops are concentrated in China, Taiwan, and Hawaii. Taro flour can be used as an ingredient for many other preparations including bread, cakes, cookies, noodles, and cereals, or even as a partial substitute for traditional whey flour [22,23,24]. 

The main carbohydrate present in taro is starch found in polygonal and small granules, averaging 1.3–2.2 µm in diameter, although granules measuring 5 µm can be observed [25]. As a starchy vegetable, taro presents part of the starch in resistant form, which can escape small intestine digestion and be directed to colon fermentation. This resistant-starch results in several health effects, including the augmented absorption of minerals, contribution in controlling blood glycemia, and reduction in plasma triglycerides and cholesterol [26].

Since taro is free of gluten and displays low protein and high calorie content, as well as low fat levels, taro consumption can benefit individuals with dietary restrictions such as those presenting allergies, especially in children and gluten-intolerant individuals, while contributing to reduce the risk of obesity and type II diabetes. In addition, the presence of soluble and non-soluble dietary fibers can improve intestinal transit and possibly aid in colorectal cancer prevention. As a result of its gluten-free nature, taro flour has arisen as a promising substitute for wheat flour, boosting the Brazilian market for gluten-free derivatives [13,15,16,27,28,29].

To encourage and reinforce the importance of taro consumption, this study aims to discuss the benefits of the biofunctional compounds found in taro in promoting health, especially considering their potential against cancer, as well as in the control of other physiopathological conditions that compose the risk factors for cancer burden, including obesity and type II diabetes. 

### 1.3. Risk Factors Associated with Cancer and the Impact of Healthy Dietary Habits

Cancer, obesity, and type II diabetes are the main causes of mortality and morbidity worldwide. Over the past decades, the prevalence of these diseases has increased considerably in most countries, as high body mass index and diabetes are responsible for 5–7% of all cancers, which is equivalent to 804,100 new cases in 2012 [30].

The risk of cancer is directly associated with both intrinsic and non-intrinsic factors. Intrinsic factors are inherent to human cell biology and, therefore, non-modifiable, such as spontaneous modifications in DNA that can contribute to the cancer initiation process. Non-intrinsic elements are entirely modifiable and comprehend endogenous and exogenous aspects of cultural habits acquired by society, such as unhealthy lifestyles, exposure to carcinogenic/mutagenic chemicals, and the generation and accumulation of free radicals throughout life [31].

Concerning personal individualities, endogenous agents may affect cell proliferation mechanisms and genome integrity triggered by biological aging, genetic susceptibility (heritable cancer genes), hormone and growth factor levels, metabolic dysfunctions, chronic inflammatory status, oxidative stress, and compromised immunological competences. Exogenous agents contribute to about 70% risk of cancer development compared to endogenous factors, by causing new mutations, the activation of oncogenes, or the inhibition of tumor suppressor genes [32]. Exogenous agents include infectious diseases that can be prevented by vaccination, protected sex, and no needle sharing. Among contagious diseases, *Helicobacter pylori* is associated with gastric cancer, human papillomavirus (HPV), with cervical, head and neck cancers, and hepatitis virus B and C, with hepatocellular carcinoma. Mutagens/carcinogenic agents comprise environmental physical or chemical agents such as UV radiation, air pollution substances (benzene, radon), and tobacco (containing 69 types of carcinogenic chemicals), which, if avoided, can prevent DNA damage. Many other substances are related to specific work environments (wood dust, nickel compounds), secondhand tobacco smoke, food (aflatoxins), and hormone therapy (estrogens), and they should be avoided or minimized when possible. Other exogenous agents can be grouped in the lifestyle category, including smoking, alcohol, and dietary habits [31]. 

A healthier lifestyle concept includes physical activity and adequate nutritional habits while excluding smoking and alcohol consumption. Chronic inflammatory diseases linked to dietary habits such as type II diabetes and obesity are among the exogenous risk factors frequently associated with cancer cases [33,34]. On the other hand, a diet including vegetables, fruits, and greens is highly associated with a reduced risk of cancer development in humans [31,35,36,37]. These food sources are widely known to be rich in bioactive compounds and displaying pharmacological properties such as antioxidant, anti-inflammatory, anti-obesity, anti-diabetes, immunomodulatory, antimetastatic, and antitumoral activities which, together, can contribute to delay cancer burden, since they prevent oxidative stress, modulate the proinflammatory status, and control metabolic dysfunction [33,38]. Several of those pharmacological bioactivities can be found in taro (*Colocasia esculenta* [L.] Schott), which is a highly nutritious food that is capable of preventing starvation and malnutrition in countries below the poverty line [5]. 

## 2. Bioactive Compounds and Pharmacological Properties of Taro−Popular Medicinal Knowledge with Anticancer Potential 

The use of taro to treat multiple unhealthy conditions and diseases such as diabetes, hemorrhage, diarrhea, arterial hypertension, alopecia, among others, dates from ancient times [39,40].

Studies on *Colocasia esculenta* have been conducted worldwide, but especially in geographic regions where taro cultivation is notably expressive, such as the USA, Nigeria, South Africa, Samoa, Korea, Japan, Philippines, Bangladesh, Indonesia, and New Zealand (Figure 1 and Table 2). Taro’s health-promoting potential has been confirmed by in vitro and in vivo preclinical assays, by assaying raw or cooked corms and taro derivatives in the form of flour or extracts (Table 2) [41,42,43,44,45,46,47,48,49,50,51,52,53,54,55,56,57,58,59,60,61,62,63]. 

The bioactivities found in taro have been mainly attributed to its polyphenols, proteins, mucilage, polysaccharides, lipids, and non-polyphenol antioxidants. Many of these bioactive principles have already been identified and singly assayed, proving their participation in these claimed activities. Bioactive molecules identified in taro include tarin, taro-4-I polysaccharide, taro polysaccharides 1 and 2 (TPS-1/TPS-2), A-1/B-2 α-amylase inhibitors, monogalactosyldiacylglycerols (MGDGs), and digalactosyldiacylglycerols (DGDGs). Together, these data clearly indicate that the biological effects exerted by taro are possibly a synergic effect of multiple compounds displaying effectiveness not only against several cancer cell lines but also against some of the main external cancer risk factors, such as free radicals, mutagenic and carcinogenic agents, and physiopathological conditions such as obesity and type II diabetes (Table 2). 

Interestingly, many of the bioactivities were demonstrated with cooked taro formulations, even following oral administration, suggesting that the recommendation to include taro in the daily human diets, alongside other healthy eating habits, could contribute to the efforts to reduce cancer risks.

### 2.1. Taro Antioxidants 

Antioxidants, under appropriate concentrations, protect, prevent, or delay the oxidation of biomolecules such as nucleic acids (DNA and RNA), protein, and lipids [64]. Foods enriched in antioxidants may contribute to health maintenance, especially concerning comorbidities caused by oxidative stress elicited by an excess of oxygen/nitrogen reactive species [65,66]. 

Reactive species may be produced in physiological conditions, through aerobic metabolism and macrophage activation, or in pathological states following exposure to xenobiotics, such as toxins, pollutants, cigarettes pesticides, or radiation. To counterbalance oxidative stress, phytochemicals with antioxidant activity found in foods such as fruits, vegetables, cereals, and tubers, are noteworthy as a relevant diet intervention topic. Evaluating the total antioxidant capacity (TAC) of foods is useful to improve functional diet quality and assist in health maintenance [67,68]. 

Many phytochemicals found in taro display the potential to reduce oxidative stress. The H-ORAC (Hydroxyl Radical Antioxidant Capacity), ABTS (2,2′-Azino-bis[3-ethylbenzothiazoline-6-sulfonic acid] diammonium salt), FRAP (ferric reducing antioxidant power), and DPPH (2,2′-diphenyl-1-picrylhydrazyl radical assay) methods have been applied to evaluate the TAC of taro extracts or taro-food derivatives. Flavonoids, tannins, saponins, alkaloids, carotenoids, phenols, vitamins, and fatty acids seem to contribute to overall taro antioxidant capacity (Table 3) [13,52,53,69,70,71,72,73,74,75,76,77,78,79,80,81,82,83]. 

Several studies point out distinct antioxidants in variable amounts in taro and taro derivatives and these differences might be attributed to multiple factors, such as (*i*) genetic background between variants of the same species; (*ii*) the applied farming cultivation system; (*iii*) post-harvest processing, including the handling, cutting, peeling, drying, cooking, and storage stages; and (*iv*) non-standard extraction of bioactivities [84,85,86,87,88,89].

Few studies go beyond TAC determination and phytochemical group characterization to identify specific compounds within the aforementioned class, and at what concentrations, in taro [69,70,74,76,78,80]. The taro matrix presents a complex set of antioxidants and, although they have been identified and measured, it is hard to forecast which role a single compound will play on the human body. Taro antioxidants may function in combination to promote synergistic or antagonistic effects [90]. 

Chlorogenic acid, catechin, epicatechin, epigallocatechin, pro-anthocyanidins, and gallic acid have been identified in taro by thin-layer chromatography (TLC) and high-performance liquid chromatography (HPLC) [69]. Polyphenols represented by 1-O-feruloyl-D-glucoside; 3, 5-DiCQ acid; vitexin; isovitexin; cyanidin-3-glucoside; luteolin-7-O-ruti-noside; vicenin-2; caffeic acid; cyanidin-3-rhamnoside; chlorogenic acid; quercetin and hyperoside were identified by LC-MS (liquid chromatography-mass spectrometry) [76].

**Figure 2:**
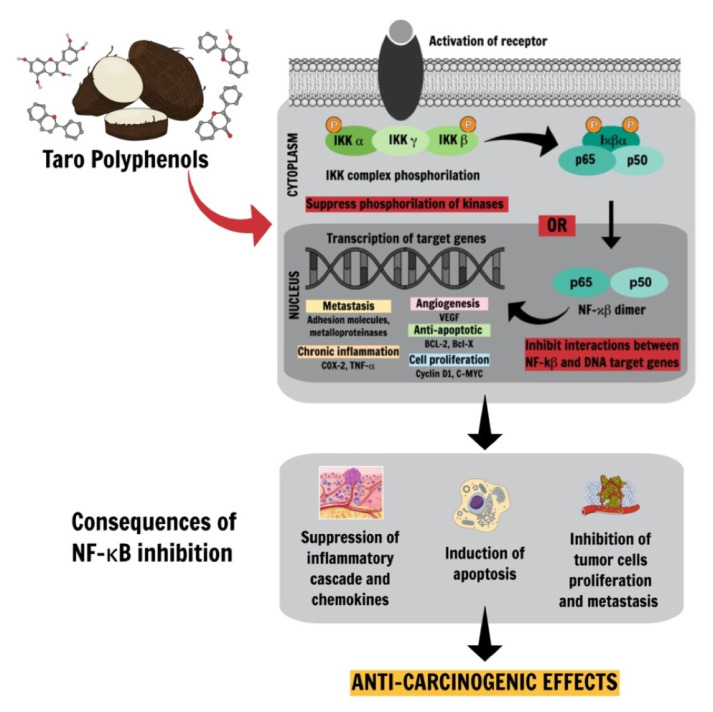
Therapeutic potential of taro phytochemicals: Anti-carcinogenic effects of taro bioactive compounds on the non-canonical NF-κB pathway could be mediated by polyphenols. Polyphenols can mediate the suppression of NF-κB transcriptional factor resulting in the inhibition of pro-inflammatory signaling cascade, apoptosis induction, cell proliferation control, and metastasis. inhibitory kappa B kinase (IKK); vascular endothelial growth factor (VEGF); B-cell lymphoma 2 (BCL-2); B-cell lymphoma X (BCL-X); c-myelocytomatosis (C-MYC).

Polyphenols and other antioxidants present in taro, besides acting as common free-radical scavengers, other molecular and enzymatic mechanisms triggered by polyphenols, are considered complementary for health promotion. Few mechanisms of action delineate how polyphenols participate in inflammatory cascades by increasing pro-inflammatory cytokines release. Subsequently, inflammation can, for example, activate the NF-κB (nuclear factor kappa B) transcription factor and stimulate the production of TNF-α, two critical factors that, when upregulated, may participate in cancer development. Thus, polyphenols could aid in controlling cancer progression due to their anti-inflammatory effects, including the activation of antioxidant enzymes, inhibition of pro-oxidant enzymes, and prevention of free radical attacks [91] (Figure 2). 

Under normal conditions, NF-κB is held inactive in the cytosol by the inhibitor of NF-κB proteins, inhibitor kappa B (IκB) (α, β, ε). Upon binding to different immune receptors, the IκB kinase complexes phosphorylate IκB and thereby induces its proteasomal degradation and the release of NF-κB. The released transcription factor NF-κB can translocate to the nucleus and mediates the expression of target genes. Polyphenols may act either by suppressing kinases complex phosphorylation, which avoids NF-κB translocation to the nucleus or inhibits the interaction of this transcription factor with targeted DNA genes. Both mechanisms can down-regulate the inflammatory cascade, inducing apoptosis, controlling both cell proliferation and metastasis [92]. 

Food processing, such as cooking, frying, steaming, fermentation, and other conservation methods, including freezing, pasteurization, and drying, interfere in antioxidant concentrations, releasing more antioxidants, as observed with phenolic acids bound to complex structures such as lignin and cellulose [93,94]. Freeze-dried foods can preserve the antioxidant compounds [95]. 

Lipid-soluble antioxidants found in taro have been identified by HPLC. Vitamins and carotenoids, among them ascorbic acid, violaxanthin, lutein, β-carotene, δ-γ-α-tocopherol, and δ-γ- tocotrienol are responsible for protecting cellular structures against lipid peroxidation caused by free radicals (Table 3) [41,51,62].

Several fatty acids present in taro are suggested as potential antioxidants, including 9,12,15-octadecatrienoic acid, 8,11-octadecadienoic acid, methyl ester; hexadecanoic acid, methyl ester; 9-octadecenoic acid, methyl ester (E); 3,5-Di-tert-butyl-4-trimethylsiloxytoluene; cyclohexanol, 2-nethyl-5-(1-methylethenyl)- (1. alpha., 2. beta., 5. alpha.) [74,78]. 

Despite positive results, recent studies have indicated that reactive oxygen species (ROS) scavenging in cancer cells, particularly during chemotherapy, interferes with the primary mechanism, which triggers apoptosis in these cells. In this way, antioxidant supplementation is a double-edged sword. To be used as a supportive therapy during cancer treatments, it might take into account the type of cancer, general patient status, and antioxidant dosage and type to reach the best performance. Another suggestion is to administer a single antioxidant prescribed by a unique kind of cancer, since antioxidants, as mentioned previously, can play synergic or antagonistic effects and even lead to uncontrollable effects in the human body when administrated as a non-controlled combination [96].

The data in Table 3 summarize the available studies concerning the antioxidant capacity of taro per geographic region, as well as the compounds supposed to be involved in the activity. Most investigations have been carried out in Asia, Oceania, and Africa, where taro plays a vital role as a staple food. However, the restricted distribution of taro-related studies reinforces the claims that taro is still a neglected crop worldwide, even though it displays a rich composition concerning health-influencing compounds (Figure 1) [97]. In the beginning of the 1980s, complaints about the lack of research efforts focused on taro were noted. At that time, taro was considered the most underrated root crop in a number of reports and studies [98,99]. Nowadays, this number has increased, and more science institutes are carrying research on taro and its potential benefits for human health, although it is still concentrated in under-developed areas and not worldwide, as it should be. In the next section, each traditional claimed health benefit was harbored in the experimental evidence observed in cell cultures or animal models.

### 2.2. Taro Protection against Mutagenic and Carcinogenic Agents 

**Figure 3:**
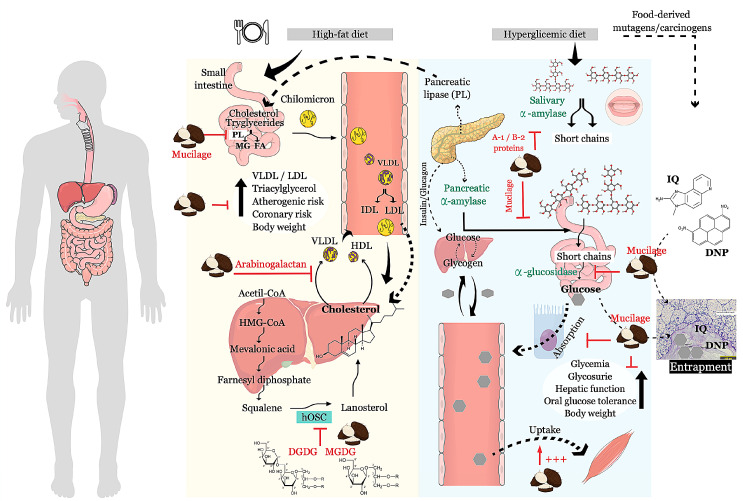
Putative targets of taro-derived components in metabolic pathways. Lipid and carbohydrate metabolisms can be modulated, mainly, by taro mucilage, arabinogalactan, monogalactosyldiacylglycerols (MGDG), digalactosyldiacylglycerols (DGDG) and A-1/B-2 proteins, which may act in conjunction to control glycemia, lipidemia, and downstream effects such as body weight, glucose tolerance, and glycosuria, hepatic function, atherogenesis, and coronary risk. Inhibition of human lanosterol synthase (hOSC) affects the cholesterol synthesis of pancreatic lipase (PL), reducing triacylglyceride hydrolysis to monoglycerides (MG) and free fatty acids (FA), down-regulation of salivary α-amylase, glucose release by α-glucosidase, glucose absorption, reduction of very low-density lipoprotein (VLDL) formation, and enhancement of muscle glucose uptake. Mucilage can entrap mutagenic/carcinogenic agents, 1,8 dinitropyrene (DNP) and heterocyclic amine 2-amino-3-methylimidazo[4,5-f] quinoline (IQ), avoiding their absorption and consequent effects. HMG-CoA—β-Hydroxy β-methylglutaryl-Coenzyme A; IDL—intermediate-density lipoprotein.

Antimutagens or anticarcinogens can decrease or even prevent the deleterious effects of physical, chemical, and biological agents that cause genome modifications or mutations, triggering the carcinogenesis process when a mutation occurs in somatic cells (Figure 3). A variety of plant compounds exhibit this ability, including those found in taro such as antioxidant molecules belonging to polyphenol classes or not, dietary fiber, luteoline-derivatives, gallic acid, vitamins (ascorbic acid, b-carotene, and a-tocopherol), anthocyanins, catechins, flavonoids, and others [100,101,102]. The antimutagenic mechanism is diverse and can occur in the extra or intracellular compartments. Extracellular mechanisms include the inhibition of mutagen uptake, complexation, and/or deactivation, inhibition of endogenous mutagen formation, and favoring the absorption of protective agents. Intracellular mechanisms involve the blocking of reactive species, transmembrane transport modification, metabolism modulation, DNA metabolism and repair modulation, signaling pathway regulation, apoptosis enhancement, genomic stability maintenance, and trapping and detoxification stimulation in non-target cells [102].

The National Cancer Institute (https://www.cancer.gov), the American Institute for Cancer Research (https://www.aicr.org/cancer-prevention/healthy-eating/), and the American Cancer Society (https://www.cancer.org) recommend the intake of dietary fibers, which are found in vegetables and fruits, since their protective effects against gastric and colorectal cancer risks have been demonstrated by several reports, including meta-analysis of case-control and cohort studies [103,104,105,106,107,108,109]. The protective mechanism of dietary fibers depends on their composition, and it can be attributed to the reduced time of intestinal tissue exposure to carcinogens and short-chain fatty acids generated from fermentation by gut microbiota [110,111,112]. 

Dietary fibers found in crude taro are mainly composed of neutral monosaccharides, uronic acid, galacturonic acid, glucuronic acid, and neglectable concentrations of lignin and no starch, and they seem to be involved in protection against chemical and physical mutagenic agents (Figure 3). Crude taro extract, rich in dietary fibers, can adsorb the hydrophobic compound 1,8 dinitropyrene (DNP), which is a mutagenic and carcinogenic pollutant found in the environment, making it ineffective in intestinal cells (Table 2) [61,113]. Certainly, absorbing mutagenic compounds can reduce the risk of gastrointestinal tract cancers. Nevertheless, cancer risk reduction by dietary fiber intake can be extended to several organs, i.e., breast, pancreas, and prostate [114].

Other evidence do not ascribe antimutagenic activity to taro dietary fibers, attributing it to different compounds in taro. A heptane extract from cooked taro prevented the deleterious mutations caused by the heterocyclic amine 2-amino-3-methylimidazo[4,5-f]quinoline (IQ), which is a potent mutagenic and carcinogenic agent formed during meat and fish cooking [63]. Similarly, an aqueous extract from crude taro obtained from two different cultivars, a traditional one and the Ebi-taro from Kyoto (JPN), displayed antimutagenic effects against physical agents when assayed in *E. coli* cells exposed to UV radiation [62].

### 2.3. Anticancer, Anti-Inflammatory, and Immunomodulatory Effectiveness of Taro Extracts or Their Components

Antitumoral and antimetastatic bioactivities associated with immunomodulatory effectiveness can be provided by the set of molecules found in taro, as previously demonstrated by in vivo and in vitro preclinical trials. A soluble extract from poi (cooked taro), a popular food traditionally consumed by Hawaiians, inhibits the proliferation of rat YYT colon cancer cells in a dose-dependent manner, reaching its maximum effect at 25 mg poi/mL. Rat YYT cells displayed morphological apoptosis characteristics, confirmed by TUNEL (TdT-Mediated dUTP Nick End Labeling) nucleus staining, which is indicative of DNA damage. In contrast, no toxicity to healthy mice spleen cells was observed, reinforcing suitability for antitumorigenic use. Moreover, splenocytes were stimulated to proliferate, displaying a TCD4 phenotype, followed by TCD8, B, and natural killer (NK) cells [41]. The presence of these cells at the tumor microenvironment is a good prognosis, especially during the early stages of carcinogenesis. CD8+ T lymphocytes differentiate into cytotoxic cells by antigen-presenting cell (APC) activation, in order to promote the direct destruction of cancer cells. CD4+ T lymphocytes contribute to the fight against cancer by the release of pro-inflammatory cytokines IL-2, TNF-α, and INF-γ, which, in turn, activate T lymphocytes, NK, and macrophage cells, while enhancing antigen presentation [115]. Poi antitumoral activity may be due to both a direct effect of its bioactive compounds on cancer cells associated with an indirect effect on immune system activation. 

Crude taro extract inhibits the in vitro proliferation of several human breast cancer lines, i.e., MCF-7, MDA-MB-231, and MCF10A, as well as murine breast cancer lines 66.1, 410.4, and EpH4. The antiproliferative effects on murine cells were accompanied by morphological changes such as cell rounding and a reduction of foot projections. Crude taro extract displayed antimetastatic effects following intraperitoneal administration, before and after the establishment of cancer, exerting therapeutic and protective effects against heart and lung colonization by breast cancer lineages [42]. 

The anticancer effects promoted by taro extract have been attributed to a lectin named tarin (PDB id. 5T1X and 5T20). This protein is able to reproduce the anticancer and antimetastatic effects of the crude extract when assayed in vitro and in vivo by inhibiting human hepatoma cells (HepG2 lineage) proliferation as well as lung and heart colonization [42,44].

Tarin nano-encapsulated in liposomes improved anticancer activity compared to the free protein against human breast cancer (MDA-MB-231) and glioblastoma (U87 MG) lineages, resulting in 41 and 65% inhibition, respectively. Nano-encapsulated tarin was as effective as cisplatin and temozolomide in controlling glioblastoma cell proliferation. However, the advantage relies on the non-cytotoxicity of both forms of tarin, free or nano-encapsulated, in effective concentrations when added to healthy cells [44]. 

These findings indicate that tarin exhibits high potential as a supportive anticancer therapy [43,44]. Moreover, tarin is considered a relatively stable protein, maintaining activity under a wide range of pH and temperatures, which are mandatory physicochemical features for candidate molecules for pharmaceutical purposes [116].

This lectin has become a notorious molecule after being extensively studied and fully characterized as an anti-tumoral and immunomodulatory effector [2,48,116,117]. Not surprisingly, glycan microarrays indicate that this protein binds to specific glycan chains that are part of antigens found in many types of cancer cells, such as CA-125 in ovarian cancer, paucimannose in human cancerous cells, Lewis-y epitope, a typical antigen found in the colon, stomach, ovary, breast, pancreas, prostate and lung cancer, hematopoietic progenitor cells, peripheral blood granulocytes, and to the antigen H2, which is found in leukemic cells and hematopoietic progenitors [116]. Although binding recognition has not yet been tested in culture cells, these data profoundly reinforce that tarin may contribute to the antiproliferative, antimetastatic, and immunomodulatory responses observed upon taro extract intake as aforementioned and further discussed. 

Taro extracts have also demonstrated anti-inflammatory activity against human and murine breast cancer cells by modulating the immune response through the reduction of prostaglandin E2 (PGE2) release accompanied by the abolishment of mRNA synthesis of cyclooxygenase-2 (COX-2), and decrease of COX-1 [42]. The tarin down-regulation of COX-2 gene depressed E-series prostaglandins by almost 70%, especially PGE2, which is an inflammatory mediator known to exert pro-tumorigenic effects, contributing to (i) the generation of additional mutations in cancer cells by stimulating the release of ROS; (ii) upregulation of a series of critical molecules such as Bcl-2, conferring resistance to apoptosis, vascular endothelial growth factor (VEGF) involved in angiogenesis, type 2 and 9 matrix metalloproteinases (MMPs), which confer invasive ability, epidermal growth factor receptor (EGFR), facilitating cell proliferation, extracellular signal-regulated kinase (ERK) and membrane proteases also involved in invasion; (iii) suppression of antitumoral response by downregulating the immune system; (iv) activation of wingless-related integration (WNT)/β-catenin pathway that favor metastasis and maintain stem cell skills; (v) activation of phosphatidylinositol 3-kinase/protein kinase B (PI3K/AKT) pathway that promotes cell proliferation and survival. As a result, the angiogenesis process, cancer cell proliferation, differentiation, and migration would be prevented [42,118,119,120]. 

Previous studies indicate that the inhibition of COX-2 resulted in an overall 70% reduction in cancer risk for breast, lung, prostate and, colon cancers, and also in cancers whose etiology is associated with several mutagenic conditions such as tobacco, alcohol, UV light, oxidative stress, and infections by viruses or bacteria [120,121]. 

The selective downregulation of COX-2 by taro lectin seems to be very promising in cancer treatment, since the reduction in PGE2 synthesis attenuates pro-tumorigenesis. Further studies are required in order to understand the molecular mechanisms behind COX-2 regulation by tarin, which can occur in different ways, considering that the COX-2 promotor region harbors multiple binding sites for enhancers, including NF-κB, β-catenin, interleukins, and Hu antigen-R, among others [120]. 

**Figure 4:**
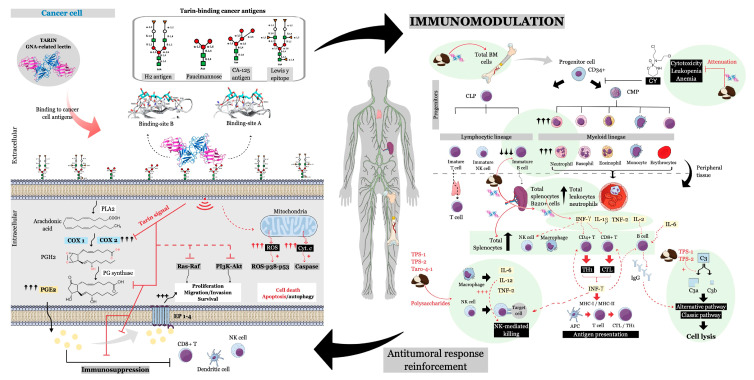
Hypothetical antitumoral tarin mechanism. (*left-hand panel*) Tarin binds to specific carbohydrate antigens, typically overexpressed in cancer cells, down-regulating COX-2 (cyclooxygenase 2) expression, culminating in decreased PGE2 (prostaglandin 2) synthesis and derepression of the antitumoral response. Tumor progression can be inhibited by inactivation of Ras–Raf or PIK3–Akt pathways and cell death by the activation of ROS-p38-p53 or caspase-dependent pathways, inducing apoptosis or autophagy, commonly described for GNA-related lectins. Red lines show already known tarin mechanisms. Dashed red lines indicate the main mechanisms reported for GNA-related lectins. PLA2—phospholipase A2; PG synthase—prostaglandin synthase; ROS—reactive oxygen species; Cyt. *c*—cytochrome *c*; EP1-4—prostaglandin E receptor 1-4; NK cells—natural killer cells. (*right-hand panel*) Anticancer effects may be boosted by the immunomodulatory abilities of tarin and other taro derivatives, whose effects are highlighted in green and include complement activation through alternative and classical pathways, natural killer (NK) cell activation, T cell activation to TCD4+ cells (Th1) and TCD8+ (CTLs—cytotoxic T lymphocytes) effectors, cytokines release by polysaccharide-activated macrophages and tarin-activated splenocytes, progenitor cell protection through the attenuation of cyclophosphamide (CY) cytotoxic effects and the proliferation of spleen and bone marrow cells. Red lines indicate tarin and polysaccharide effects, while the dotted red lines indicate the downstream effects that might be triggered by released cytokines. CLP—common lymphoid progenitor, CMP—common myeloid progenitor, BM—bone marrow.

Since tarin belongs to the *Galanthus nivalis* agglutinin (GNA)-related lectins, sharing specificity and structural characteristics with them, its mechanism of action could be extrapolated (Figure 4). Different mechanisms of action were described for these lectin members and attributed to their binding to mannose-containing antigens. These mechanisms can be linked to PGE2 overexpression, which is a typical condition in cancer. GNA-related lectins exert their cytotoxic activities on cancer cells mainly by deactivating rat sarcoma-rapidly accelerated fibrosarcoma (Ras–Raf) and PI3K–Akt pathways previously activated by PGE2 autocrine action through the prostaglandin E receptor 1-4 (EP1-4) receptor, inhibiting the proliferation, migration/invasion, and survival of cancer cells. Once the anti-apoptotic pathways are inhibited, cell death can be induced through apoptosis or autophagy triggered by mitochondria injury following the accumulation of ROS and cytochrome *c* release, culminating in the activation of ROS-p38-p53 and caspase-dependent pathways [122,123,124].

Taro components are traditionally known to boost the immunological response, which could be an indirect way to contribute to reducing cancer risk or controlling tumorigenesis (Figure 4). Indeed, in mice, tarin proved to stimulate the in vitro and in vivo proliferation of bone marrow (BM) and spleen cells while protecting BM progenitor cells from death. B lymphocytes and granulocytic cells were identified among the proliferating cells when taro extract was intraperitoneally administered (1 mg/animal). Moreover, tarin-sensitized splenocytes were stimulated to release IL-2, IL-1b, TNF- α, and INF- γ, which are essential cytokines involved in the anticancer response, as mentioned previously [86,88,96,97].

Considering that tarin is found in the edible part of *C. esculenta*, the regular intake of this tubercle could be a useful dietary intervention to boost the immune system in healthy individuals or to accelerate the recovery from leukopenia in immunosuppressed patients, including those under chemotherapy. Tarin immunomodulatory potential has been evidenced by the administration of the purified protein (200 µg/animal) to cyclophosphamide-immunosuppressed mice, where BM cells were stimulated to proliferate and differentiate into granulocytic cell lineage, promoting a faster recovery from leukopenia. Tarin also protects the BM erythroid progenitors from the cyclophosphamide cytotoxicity [50]. 

In addition to tarin, taro contains a mixture of other anti-neoplastic molecules, such as polyphenols and polysaccharides, as mentioned previously [125,126]. An ethanolic extract from freeze-dried taro exhibited an antiproliferative effect against adult T-cells leukemia lineages and, in some of them, effectiveness was superior to the apoptotic effect of genistein, an isoflavone found in soy, which is a popular grain recognized to trigger apoptosis in estrogen-dependent cancers [46,127]. A water-soluble 200 kDa polysaccharide (taro-4-I) composed of neutral sugar and uronic acid isolated from taro crude extract was able to inhibit mice lung colonization by B16BL6 melanoma cells [45].

Similar to the tarin modulatory effect, polysaccharides extracted from taro also enhanced the immune response. Taro-4-I, as well as TPS-1 and TPS-2, was able to activate the complement component 3 (C3 protein) through classical and alternative pathways, which is a prerequisite to trigger cellular lysis. In addition, macrophages and NK cells were activated, while macrophages were stimulated to release IL-12, IL-6, and TNF-a cytokines and nitric oxide (NO) [45,47].

### 2.4. Taro Compounds Effect on Type II Diabetes and Obesity

Obesity or overweight is the primary risk factor for developing type II diabetes. Diabetes, obesity, hyperglycemia, and hypercholesterolemia, occurring together or independently, have been directly associated with cancers in the kidney, bladder, thyroid, ovary, breast, endometrium, stomach, liver, pancreas, colon, and rectum, as well as leukemia. The adoption of a healthier lifestyle is the recommended strategy to prevent overweight or obesity and avoid cancers related to these comorbidities [128,129,130]. Taro can be a powerful ally considering its medium glycemic index, since 33% of total taro starch is composed of SDS and RS after cooking, and high dietary fiber content, which are two essential properties to manage these metabolic dysfunctions (Table 1). Moreover, other taro bioactive molecules can synergistically reinforce the metabolic effects of taro, as demonstrated in preclinical trials (Figure 3 and Table 2).

Taro flour offered to streptozotocin (STZ)-induced hyperglycemic rats restored glycemia after four weeks of intake. Proteinuria and glucosuria, kidney function, relative kidney weight, hepatic function, glycated hemoglobin, and body overweight, which are associated with type II diabetes, were also attenuated following taro consumption. Plasmatic levels of total cholesterol, VLDL- and LDL-cholesterols, triacylglycerol, serum pancreatic lipase, atherogenic, and coronary risk were all reverted, and the HDL-cholesterol levels were enhanced [52,53,54]. Similar effects were obtained by the administration of an ethanolic extract from crude taro flour, which decreased glucose tolerance and glycemia in a murine model [55]. The anti-hyperglycemic and anti-hyperlipidemic effects observed herein point out that taro may have the potential to manage diabetes and obesity. These effects are mainly attributed to flavonoids, alkaloids, saponins, steroids, and tannins, but the participation of minerals (Mg, Ca, K, P, Fe, and Zn) and crude dietetic fiber have not been discarded [53,55]. 

Taro mucilage, mainly composed of neutral sugar, absorbs mutagenic/carcinogenic agents and also takes part in anti-hyperglycemic and anti-hyperlipidemic effects. A mucilage-rich extract from crude taro flour inhibits the starch-hydrolytic enzymes α-glucosidase and α-amylase, and pancreatic lipase. [56,131]. Arabinogalactan extracted from taro flour mucilage and incorporated into hypercaloric rat diets decreased lipid levels in serum and tissues, and it reduced hepatocyte synthesis/secretion of apoB-containing lipoproteins, mainly VLDL [60]. The viscous mucilage fibers are known to reduce the bolus motility through the gastrointestinal tract and, as a consequence, the increasing digestion and absorption of macronutrients, especially lipids and carbohydrates, promoting satiety [132]. Glucose entrapment by plant mucilage is a widely described phenomenon [133,134,135]. 

The polysaccharide matrix can retain and deliver many bioactive compounds, such as phenolic compounds and peptides, which are both detected in mucilage-rich extracts from crude taro flour [56,131]. The importance of phenolic compounds in the management of type 2 diabetes has also been widely reported [136,137,138]. Flavonoids, phenolic acids, and tannins are generally associated with the reduction of starch digestion due to the inhibition of α-glucosidase and α-amylase activities, as mentioned previously. In the end, a reduction in glucose release and absorption can occur, controlling post-meal glycemic levels. Lipid digestion may also be regulated by phenolic compounds through the inhibition of pancreatic lipase, followed by excretion in feces, consequently reducing body mass. Both effects have been demonstrated in clinical trials, being considered adequate for diabetes and obesity control, since they share similar mechanisms of action with many drugs currently in use [119,122,123].

Additionally, proteins A-1 and B-2, with molecular masses of 17 and 19 kDa, respectively, obtained from defatted flour of crude taro, inhibit α-amylase from human saliva and porcine pancreas in vitro, thus being potentially able to control diabetes and obesity [57]. The inhibition of starch-hydrolytic enzymes by taro compounds is especially important to prevent or minimize the digestion of the rapidly-digestible starch (RDS) portion that accounts for 67% of the total starch and contributes to the sudden increase of glycemia [13].

The anti-hyperlipidemic activity exerted by taro could be explored as a source of natural inhibitors of human 3-hydroxy-3-methyl-glutaryl coenzyme A (HMG-CoA) reductase, which is the key point enzyme in cholesterol synthesis and the molecular target for drugs used to treat hypercholesterolemia. The continued use of such drugs by healthy individuals for hypercholesteremia prevention or long-term treatments may cause side effects. Based on this, the search for natural inhibitors was focused on enzymes downstream to HMG-CoA reductase, such as lanosterol synthase. From an extensive list of 130 plant extracts, one obtained from freeze-dried taro was able to cause a 55% inhibition of lanosterol synthase activity, which is an impressive result considering that less than 32 extracts showed a very slight effect, under 5% inhibition. Eight lipids purified from taro extract, three of them classified as monogalactosyldiacylglycerols (MGDGs), and five as digalactosyldiacylglycerols (DGDGs), inhibited in vitro lanosterol synthase activity by 28–67%. The therapeutic potential of MGDGs and DGDGs should be further explored in preclinical studies, since similar lipids isolated from microalgae have already been reported as inhibiting tumor-promoting agents [59].

The anti-hypercholesterolemic effect of tiwul, a traditional Indonesian dish prepared from cooked taro flour, has been demonstrated in rats. Hypercholesterolemic rats exhibited a 36% reduction in total cholesterol following two weeks of tiwul intake [58].

Taro should be included in the earliest ancient crops in the world, because the first evidence of taro consumption is dated between 28,700 and 20,100 years BP in Salomon Islands, Oceania, where taro starch remains were found in stone tools possibly used to cut raw corms, indicating that taro may have been consumed at Kilu Cave, and probably it was part of the prehistoric diet [139]. Over time, these populations have identified several health benefits from consuming taro, many of which are empirical observations that were confirmed in animal models and/or in cell lines testing for its effectiveness and no toxicity to healthy tissues. However, to date, there is a scarcity of clinical studies leaving gaps in the translation of the positive health effects identified in preclinical studies to human beings. The better exploitation and understanding of taro bioactivities, following dietary interventions in humans could be helpful in developing new functional compounds. 

Clinical trials on healthy non-diabetic young adults evidenced that taro shows a medium glycemic index, low glycemic load, and moderate glycemic response [140]. Moreover, the addition of other food components, such as vegetables, oils, and rich protein food, during cooking, can reduce the taro glycemic index to a lower rate [141]. 

Taro is reported to have anticancer potential through several preclinical analyses, as aforementioned. The Japanese population traditionally consumes starchy roots, such as taro, that are associated with a decrease in the risk of kidney cancer death [142,143,144]. 

Taken together, the intake of taro or their derivatives can provide bioactive compounds capable of promoting health benefits, especially in the control of hypercholesterolemia, which is not only a complication of diabetes and overweight but also a risk for cerebrovascular and cardiovascular diseases, which are important causes of death worldwide, along with cancer [145,146]. Moreover, the overall benefit against type II diabetes and obesity could certainly aid in reducing the risk factors for cancer. 

## 3. Conclusions

Although neglected, taro is a valuable source of several health-promoting compounds, such as taro lectin or tarin, bioactive-complex carbohydrates, and natural polyphenols and other antioxidants. In general, these molecules act through individual or synergic pathways and play a role in the modulation of cellular proliferation, differentiation, apoptosis, angiogenesis, and invasion of cancer cells. The antiproliferative and apoptosis-inducing activities in tumorigenic cells are still poorly understood, despite the evidence obtained from in vitro and in vivo assays. The bioactive compounds from taro may interact with plasmatic cell membranes and intracellular receptors, interfere with signaling cascades, regulate enzyme activities, interact with oncogenes and oncoproteins, and bind to gene promoter sequences. The result of such vast interactions can contribute to ameliorate systemic health status by managing oxidative stress imbalance, reducing systemic inflammation, modulating metabolic dysfunctions, and boosting the immune response. The molecular mechanisms of taro active compounds are not yet fully understood, which is probably due to the few studies on taro, which are restricted to specific regions around the globe, as shown along with this review. Further investigation is necessary to determine which signaling pathways are stimulated to achieve cellular immunity through the activation of T lymphocytes, promoting the apoptosis of tumor cells, how macrophages are activated and progenitors protected, as well as how taro bioactivities can modulate gene expression. Thus, many mechanisms remain to be elucidated to better exploit taro extracts, taro derivatives, or individual taro components. The non-toxicity of these molecules toward healthy cells turns taro components into potential candidates for supportive target therapies when associated with traditional drug treatments. In addition, since taro is a food matrix rich in bioactive compounds, spreading its benefits worldwide may enhance its consumption and consequently production while resulting in better population health maintenance.

## 4. Methodology 

This review article collected data regarding the anticancer potential of taro corms components in order to show the importance of including this crop in human diet and includes taro as a promising source of therapeutic agents, with especial attention to tarin. Scientific data were freely collected and followed four main steps: (i) guiding questions formulation; (ii) database searching; (iii); eligibility; and (iv) inclusion.

### 4.1. Guiding Questions

The data search was guided by the main questions:

1. What are the biological activities associated with taro corms?
2. What are the bioactive compounds responsible for taro corm health-promoting effects, and what is their mechanism of action?
3. What is the impact of the cooking process on the presence of nutritional components and bioactive compounds of taro corms?
4. How can taro corm bioactive compounds contribute to cancer fighting or prevention?
5. What could be the benefits provided by taro corm consumption?

### 4.2. Databases, Descriptors, and/or Keywords

A literature search was performed using certain keywords at Scopus (https://www.scopus.com/home.uri?zone=header&origin=searchbasic), PubMed (www.ncbi.nlm.nih.gov/pubmed), and Google Scholar (https://scholar.google.com.br), using the advanced option. 

The main keywords used in the search were: “*Colocasia esculenta*” OR “taro” OR “poi” combined or not to “tuber” OR “corm” OR “extract” OR “tarin” OR “antitumoral activity” OR “immunomodulatory activity” OR “antidiabetic” OR “anti-hyperlipidemic” OR “antimutagenic” OR “anti-hyperglycemic” OR “anti-hypercholesterolemic” OR “antioxidant”

Duplicated studies were excluded before eligibility criteria application and references of eligible studies were carefully analyzed in order to obtain additional information not covered by the primary search.

### 4.3. Eligibility Criteria for Each of the Articles Consulted

Articles found according to the search methodology were primarily selected based on titles and abstracts. When these were not enough, the entire study was carefully read.

Articles were excluded when presenting:

1. Biological activities not directly related to cancer fighting or prevention and cancer risk factors (antimicrobial, anti-insect, antiviral, anti-helminthic, and others) for this study;
2. Studies performed with parts of the plant other than the corm, such as leaves, petioles or roots;
3. Unclear or wrong data.

Criteria used to include articles in the present review should comprise:

1. Experimental studies (in vitro, in vivo or clinical trial) that analyzed biological properties considered important for cancer fighting or prevention, such as antioxidant, antitumoral, antimetastatic, immunomodulatory, anti-hyperglycemic, antidiabetic, antimutagenic, and anti-hyperlipidemic activities;
2. Studies performed with corms, edible part of taro;
3. Articles published up to 2020 in the English language with no restriction regarding time period;
4. Review articles describing *Colocasia esculenta* characteristics, production, nutritional importance, medicinal uses and other general information;
5. Studies that purify or identify any taro component that has been proven to exhibit the claimed biological activities specified in item 1.

## Figures and Tables

**Table 1:** Nutritional composition of taro analyzed raw, cooked and baked.

Nutritional Composition			
Principle *	Nutrient per 100 g of Dry Weight		
	Crude Taro	Cooked Taro	Baked Taro with Salt
Water	70.64 g	63.8 g	60.98 g
Energy	112 kcal	142 kcal	144 kcal
Carbohydrates	26.46 g	34.6 g	34.09 g
Protein	1.5 g	0.52 g	1.93 g
Total fat	0.20 g	0.11 g	0.26 g
Cholesterol	0 mg	0 mg	0 mg
Dietary fibers	4.1 g	5.1 g	5.3 g
Ash	1.2 g	0.97 g	na
Vitamins *			
Folates	0.022 mg	0.019 mg	0.023 mg
Niacin	0.600 mg	0.510 mg	0.734 mg
Pantothenic acid	0.303 mg	0.336 mg	na
Pyridoxine	0.283 mg	Na	na
Riboflavin	0.025 mg	0.028 mg	0.031 mg
Thiamin	0.095 mg	0.107 mg	0.110 mg
Vitamin A	0.004 mg	0.004 mg	0.005 mg
Vitamin C	4.5 mg	5 mg	4.3 mg
Vitamin E	2.38 mg	2.93 mg	3.07 mg
Vitamin K	0.001 mg	0.0012 mg	0.0013 mg
Electrolytes *			
Sodium	11 mg	15 mg	475 mg
Potassium	591 mg	484 mg	762 mg
Minerals *			
Calcium	43 mg	18 mg	56 mg
Copper	0.172 mg	0.201 mg	0.222 mg
Iron	0.550 mg	0.720 mg	0.710 mg
Magnesium	33 mg	30 mg	43 mg
Manganese	0.383 mg	0.449 mg	na
Selenium	0.0007 mg	0.0009 mg	0.0009 mg
Zinc	0.230 mg	0.270 mg	0.300 mg
Starch ** (g starch/100 g)			
Total starch	18.8	14.2	na
Resistant Starch—RS	5.2	2.1	na
Slowly digestible starch—SDS	13.6 (SDS+RDS)	2.5	na
Rapidly digestible starch—RDS		9.6	na
Glycemic Index **	na	Medium	Medium

* FoodData Central in USDA (https://fdc.nal.usda.gov/) [12]. na—Not applied ** [13,14].

**Table 2:** Protective and therapeutic potential of taro corms.

Property	Taro Corm Preparation	Bioactive Compound Class	Active Principle	Screening	Biological Effect of Its Derivatives	Reference	Origin of Taro Corms
**Antitumoral/Antimetastatic**	poi extract	-	-	In vitro	Inhibition of rat YYT colon cancer cells (nearly 80–90%)	[41]	USA
	Crude taro extract	Protein	Tarin	In vitro	Inhibition of murine breast cancer cell lines: 66.1 (54%), 410.4 (24%) and EpH4 (21%)	[42]	
					Inhibition of human breast cancer cell lines: MCF-7 (65%), MDA-MB-231 (26%), MCF10A (31%)		
					Reduction of PGE2 release (63%) and mRNA COX-1/2 expression		
				In vivo	Inhibition lung and heart colonization by murine lineages 66.1 and 410.4 (85–99% reduction)		
				In vitro	Inhibition of human hepatoma HepG2 cells (25%)	[43]	China
			Tarin nano-liposomal capsules	In vitro	Inhibition of human breast cancer MDA-MB-231 (41%)	[44]	Brazil
					Inhibition of human Glioblastoma U87 MG lineage (65%)		
		Polysaccharide	Taro-4-I	In vivo	Inhibition of lung colonization by murine B16BL6 melanoma cells (96.2%)	[45]	Korea
	Ethanolic crude taro extract	-	-	In vitro	Inhibition of various adult T-cells leukemia (ATL) lineages IC50 from 25 to 106.9 μg/mL	[46]	Japan
**Immunomodulatory**	poi extract	-	-	In vitro	Proliferation of spleen cells (T, B and NK cells)	[41]	USA
	Crude taro extract	Polysaccharide	Taro-4-I	In vitro	Activation of complement system by alternative and classical pathways	[45]	Korea
					Release of IL-6, IL-12 and TNF-α by macrophages		
					Activation of NK cells cytotoxicity against Yac-1 cells		
			TPS-1 and TPS-2	In vitro	Activation of macrophage (RAW 264.7) with releasing of NO, IL-6 and TNF-α	[47]	China
		Protein	Tarin	In vitro	Cytokine expression IL-2, IL1β, INF-γ and TNF-α	[43]	
					Total spleen cells proliferation		
					Proliferation of mice spleen cells	[48]	Brazil; Philippines; China
					Proliferation of mice bone marrow cells	[49]	
					Protection of BM mice granulocytic progenitor cells	[50]	Brazil
					Promotion of repopulation of BM granulocytic Gr1+ cells		
				In vivo	Attenuation of leukopenia in immunosuppressed mice		
					Enhancement of BM granulocytic progenitors		
					Proliferation/differentiation into mature BM granulocytes		
					Protection of BM erythroid cells from CY-cytotoxicity		
					3.3-fold enhancement on 5th day (mice spleen cells) 4.1-fold enhancement on 5th day (mice Spleen B lymphocytes)	[48]	
		-	-	In vivo	Proliferation of total mice spleen cells in 5 days	[51]	
					Proliferation of B220+ lymphocytes from mice spleen in 5 or 10 days		
					Proliferation of total mice bone marrow cells in 10 days		
				In vitro	Proliferation of total mice spleen cells		
**Anti-hyperglycemic**	Taro flour	Flavonoid; Alkaloid; saponin; tannin	-	In vivo	Decrease of blood glucose on hyperglycemic rats Amelioration of biochemical parameters in the urine Amelioration of liver, hepatic function and, body weight	[52,53,54]	Nigeria
	Methanolic extract of taro flour	Alkaloid; flavonoid; steroid			Improvement of oral glucose tolerance	[55]	Bangladesh
					Decrease of blood glucose levels (35.8%)		
	mucilage-rich extract from crude taro flour	Neutral sugar; protein, polyphenols	-	In vitro	Inhibition of alpha-amylase IC_50_ = 2.23 ± 0.24 mg/mL	[56]	South Africa
					Inhibition of alpha-glucosidase IC_50_ = 1.60 ± 0.17 mg/mL		
				Ex vivo	Inhibits glucose absorption in isolated rat jejunum IC_50_ = 1.82 ± 0.24 mg/mL		
					Enhances glucose uptake by rat psoas muscle GU_50_ = 0.78 ± 0.13 mg/mL		
	Extract from defatted crude taro flour	Protein	A-1 and B-2	In vitro	Inhibition of human pancreatic (28.5 and 48.5%) and salivary alpha-amylase (62 and 56%)	[57]	
**Anti-hypercholesterolemic or Anti-hyperlipidemic**	Taro flour	Flavonoid; Alkaloid; saponin; tannin	-	In vivo	Decrease of total cholesterol, VLDL-, LDL-cholesterol, triacylglycerol, atherogenic and coronary risk. Increase of HDL-cholesterol	[52,54]	Nigeria
					Reduction of serum pancreatic lipase levels		
	Extract from cooked taro flour	-			Reduction of total blood cholesterol (36.41%)	[58]	Indonesia
	mucilage-rich extract from crude taro flour	Neutral sugar; protein, polyphenols	-	In vitro	Inhibition of pancreatic lipase IC_50_ = 1.63 ± 0.15 mg/mL	[56]	South Africa
	Ethanolic extract from crude taro	Lipid	Extract		Inhibition (55%) of human lanosterol synthase (hOSC)	[59]	Japan
			MGDG 1-3		Inhibition (28–67%) of human lanosterol synthase (hOSC)		
			DGDG 1-4				
	Mucilage-rich extract from taro flour	Polysaccharide	Arabinogalactan	In vivo	Decrease lipid levels in serum and tissues; decrease synthesis/secretion of apoB-containing lipoproteins, mainly VLDL, by hepatocytes.	[60]	India
**Anti-mutagenic**	Dietary fiber-rich extract from crude taro	Polysaccharide	-	In vitro	Avoid mutation induced by DNP (87%)	[61]	Samoa
	Crude taro extract	-			Avoid mutation by UV radiation with ID_50_ = 0.6 mg/plate	[62]	Japan
	Heptane extract from cooked taro				Avoid mutation by IQ with IC_50_ = 200–500 μg/plate	[63]	New Zealand
**Antioxidant**	See Table 3						

(**nearly 80**–**90%**)—Calculated percentage. **NK**—natural killer. **Yac-1 cells**—mouse lymphome cell line sensitive to NK cells. **NO**—nitric oxide. **BM**—bone marrow. **CY**—cyclophosphamide. **MGDG**—monogalactosyldiacylglycerols. **DGDG**—digalactosyldiacylglycerols. **DNP**—1,8 dinitropyrene. **IQ**—heterocyclic amine 2-amino-3-methylimidazo [4,5-f ]quinoline. **PGE2**—prostaglandin E2. **COX 1/2**—cyclooxygenase 1 and 2. **TNF-α**—tumour necrosis factor alpha. **IL**—interleukin. **INF**-**γ**—interferon gamma. **VLDL**—very low-density lipoprotein. **HDL**—high-density lipoprotein. **LDL**—low-density lipoprotein. **IC50**—half maximal inhibitory concentration. **GU50**—glucose uptake. **hOSC**—human oxidosqualene--lanosterol cyclase. **apoB**—apolipoprotein B. **ID50**—inhibitory dose for 50% of relative mutagenic activity.

**Table 3:** Antioxidants in *Colocasia esculenta* from different geographic regions.

Taro Corm Origin	Taro Derivatives and Method of Extraction	Antioxidant Molecules	Methods Used for Antioxidant Evaluation	Quantification	Antioxidant Capacity	Ref.
Cameroon	Raw Taro Corms Acetone: water extraction	Polyphenols Chlorogenic acid; Catechin; Epicatechin; Epigallocatechin (flavan-3-01s); Gallic acid; Proanthocyanidins.	TLC and HPLC.	Non applicable	Non applicable	[69]
China	Raw Taro Corms Acetone: water: acetic acid extraction	Ascorbic acid; Violaxanthin; Lutein; β-Carotene; δ-γ-α-Tocopherol; δ-γ-Tocotrienol.	H-ORAC, Folin–Ciocalteu method (a) ascorbic acid determination (b) and HPLC evaluation.	(a). 1.8 mg GAE/g FW (b). 29.5 µg AA/g FW	12.96 µmol TE/g FW	[70]
Egypt	1. Fresh Taro Corm Ice Cream 2. Boiled Taro Corm Ice Cream	(a) Flavonoids (b) Tanins (c) Ascorbic acid (d) Carotenoids	Free-radical scavenging activity (RSA), total flavonoid contents and chemical composition of taro corms.	1. (a). 88 μg/100 g (b). 0.92 (c). 31.54 mg/100 g (d). 328 μg/100 g 2. (a). 73 μg/100 g (b). 1.84 (c). 29.18 mg/100 g (d). 273 μg/100 g	CControl: 25.14% 11. 45.84% 22. 44.73%	[71]
Fiji	1.Steamed corms 2. Unsteamedcorms Methanol extraction	Phenols Flavonoids	Folin-Ciocalteu method, aluminium chloride colorimetric method, DPPH, FRAP e ABTS.	TPC (mg GAE1)/g dry weight) 1. (a) 42.77 ± 3.39 2. (a) 32.32 ± 4.56 TFC (mg CE/g dry weight) 1. (a).12.68 ± 4.85 2. (a).10.24 ± 3.51	DPPH (%) 1. (a) 4.82 ± 2.91 2. (a) 24.37 ± 4.23 FRAP (mM TE) 1. (a) 339.08 ± 20.50 2. (a) 224.72 ± 13.24 ABTS (%) 1. (a) 56.34 ± 3.54 2. (a) 42.33 ± 0.31	[72]
India	Raw Taro Corms Methanol: acetone: water: acetic acid extraction	Phenols Tanins Condensed anthocyanins	Folin-Ciocalteu method, condensed tannins, total anthocyanins, DPPH, ABTS and FRAP.	TPC—0.887 ± 0.016 mg GAE/100 mg FW	DPPH—EC_50_ from 1.390 to 2.890 mg/mL	[73]
				CTC—0.015 ± 0.001 mg CE/100 mg FW	ABTS—EC_50_ from 1.720 to 2.360 mg/mL	
				TA—4.09 ± mg/100 mg FW	RPA -EC_50_ from 4410 to 5190 mg/mL	
	Raw Taro Corms Methanol extraction	Alkaloids Tanins Terpenoids Flavonoids Fatty acids 9,12,15- octadecatrienoic Acid (?) Decanoic acid (?) 10 Fluoro trimethyl Ester (?) Pentadecanoic acid	Hager’s test and Wagner’s test, Ferric chloride test, Lead acetate test, Salkowski test and tannins, TLC, GC-MS and DPPH.	Non applicable	78.73% of inhibition	[74]
	1. Raw Taro Corms from Greenhouse Plants; 2. Micropropagated Plants; Methanol extraction	Phenols	DPPH and Folin-Ciocalteu method.	1. 88.1 ± 2.0 mg/g 2. 93.5 ± 1.3 mg/g	1. from 39 ± 1.2 to 92 ± 0.7 2. from 39 ± 1.2 to 92 ± 0.7	[75]
	Raw Taro Corms Methanol extraction	Catechin Epi-catechin 1-O-feruloyl-D-glucoside 3, 5-DiCQ acid Vitexin Isovitexin Cyanidin-3-glucoside Luteolin-7-O-ruti-noside Vicenin-2-Cafeic acid Cyanidin-3-rhamnoside Chlorogenic acid Quercetin Hyperoside	Folin-Ciocalteu method, aluminium chloride colorimetric method, DPPH, FRAP, phos-phomolybdenum method and LC-MS.	TFC—10.78 mg RE/100 g	DPPH—21.80%	[76]
					TAA—63.47 mg AAE/100 g	
				TPC—14.17 mg GAE/100 g		
					FRAP—63.71 mg BHT Eq/100 g	
Japan	Raw Taro Corms Methanol/water/acetic acid extraction	Polyphenolic compound*s*	H-ORAC (a), Folin-Ciocalteu method and DPPH (b).	0.46 mg GAE/g	a. 10.10 mmol TE/g b. 2.47 mmol TE/g	[77]
	Raw Taro Corms Methanol extraction	a. 8,11-octadecadienoic acid, methyl ester; b. hexadecanoic acid, methyl ester; c. 9,12,15-octadecatrienoic acid, methyl ester (Z,Z,Z); d. 9-octadecenoic acid, methyl ester (E); e 3,5-di-tert-butyl-4-trimethylsiloxytoluene; f. cyclohexanol, 2-nethyl-5-(1-methylethenyl)-(1.alpha.,2.beta.,5alpha).	GC-MS, DPPH.	a. 54.62% b. 20.55% c. 12.06% d. 06.42% e. 01.96% f. 01.88%	Strip > Leaf > Root	[78]
Malaysia	1. Raw taro flour 2. Boiled in Water 3. Fried in groundnut oil	(a) Flavonoids (b) Tannins (c) Alkaloids (d) Carotenoid Vitamins ThiamineRiboflavin	Flavonoid and tannin contents according to AOAC, Gravimetric method, DPPH and reducing power assay.	1. (a). 0.88 ± 0.11% (b). 0.90 ± 0.00% (c). 3.68 ± 0.95% (d). 3.28 ± 0.04 μg/g 2. (a). 0.43 ± 0.32% (b). 1.83 ± 0.04% (c). 1.35 ± 0.92% (d). 1.73 ± 0.02 μg/g 3. (a). 0.38 ± 0.11% (b). 1.42 ± 0.02% (c). 1.03 ± 0.04% (d). 1.54 ± 0.02μg/g	DPPH (%) 1. 64.05 ± 2.50 2. 56.88 ± 3.77 3. 44.20 ± 3.10 Reducing power (nm) 1. 1.60 ± 0.33 2. 1.58 ± 0.37 3. 1.34 ± 0.30	[79]
Nigeria	Rat Feed Taro Corm Based	a. Flavonoids b. Alkaloids c. Saponins d. Tannins	Gravimetric method of Harbone, DPPH and TLC.	a. 2.65% b. 1.01% c. 0.70% d. 1.06%	Moderate (+++)	[53]
	Taro Corm Flour		Gravimetric method.	a. 0.64 ± 0.10% b. 0.37 ± 0.05% c. 0.51 ± 0.04% d. 0.28 ± 0.03%	Non applicable	[52]
	Taro Corm Flour Methanol extraction	a. hexadecanoic acid methyl ester; b. octadecanoic acid; c. 12-octadecadienoyl chloride; d. 11-octadecenoic acid methyl ester; e. 9-octadecenoic acid; f. 3-hexadecyloxycarbonyl-5- (2-hydroxylethyl)-4-methylimidazolium ion; g. hexanedioic acid, bis(2-ethylhexyl) ester; h. 3, 5-di-t-butyl phenol. i. flavonoids—mg quercetin equivalent/g dry weight j. Phenols—mggGallic acid equivalent/g dry weight k. Condensed tanins—mg Catechin Equivalent/g dry weight	GC–MS, polyphenol assays, total flavonoids, total condensed tannins, DPPH and reducing power assay.	a. 0.43% b. 20.91% c. 0.77% d. 2.12% e. 64.37% f. 1.36% g. 1.36% h. 3.27% i. 8.50 ± 0.42 j. 15.15 ± 0.35 k. 4.40 ± 0.14	0.58 ± 0.36 nm	[80]
	1. Raw taro corms 2. Taro powder 3. Taro-based noodles 4. Taro-based cookies	(a) Phenols*—*mg/100g expressed as g/catechin eq/g (b) Tanins*—*mg/100g expressed as vanillin eq/g (c) Flavonoids—mg/100g expressed as mg gallic acid eq/g (d) Saponins*—*mg/100g saponins	Lipid peroxidation (%), Folin-Ciocalteu method, Vanillin method, flavonoid content, saponin content.	1. (a). 34.83 ± 0.28 (b). 32.24 ± 0.35 (c). 28.56 ± 0.23 (d). 14.22 ± 0.36 2. (a). 78.33 ± 0.66 (b).32.24 ± 0.35 (c). 64.23 ± 0.54 (d). 26.96 ± 0.61 3. (a). 16.27 ± 0.90 (b). Negative (c). 2.96 ± 0.323 (d). 5.01 ± 0.61 4. (a). 3.68 ± 0.10 (b). Negative (c). 0.90 ± 0.16 (d). 2.73 ± 0.18	1. 74.68 ± 0.44% LP 2. 81.77 ± 0.47% LP 3. 65.91 ± 0.27% LP 4. 28.00 ± 0.12% LP	[81]
Philippines	1. Aqueous extraction 2. Boiled taro corms 3. Ethanol extraction 4. Hexane extraction	Non-identified	% inhibition of lipid peroxidation.	Non applicable	1. 100% 2. 100% 3. 70 ± 16% 4. 83 ± 7%	[82]
South Africa	Raw Taro Corms Methanol: water extraction	a. Flavonoids b. Phenols	Folin–Ciocalteu method, total flavonoid content, ABTS(c), DPPH (d)	a. 61 ± 9 CAE/100 g b. 205 ± 53 CAE/100 g	(c). 452 ± 72 mM TEAC/100 g (d). 244 ± 73 mM TEAC/100 g	[13]
Turkey	Raw Taro Corms Ethanolic extraction	a. Phenols b. Flavonoids	DPPH (c), ABTS (d), reducing power assay (e), Folin–Ciocalteu method and total flavonoid amount.	a. 2400 mg GAE/kg b. 2050 mg QE/kg	(c). Vitamin C > Trolox > C. esculenta > BHA—95.4, 93.6, 83.8 and 78.8% (d). Trolox = BHA > C. esculenta—100, 100 and 94.6% (e). BHA > Trolox > C. esculenta,	[83]

**TPC**—total phenolic content; **TFC**—total flavonoid content; **CTC**—condensed tannin content; **TA**—total anthocyanin content; **RPA**—reducing power assay; **TAA**—total antioxidant activity; **DPPH**—2,2′-diphenyl-1-picrylhydrazyl radical) assay; **HORAC**—Hydroxyl Radical Antioxidant Capacity; **FRAP**—ferric reducing antioxidant power; **ABTS**—2,2′-Azino-bis(3-ethylbenzothiazoline-6-sulfonic acid) diammonium salt; **TLC**—Thin-layer chromatography; **HPLC**—High performance liquid chromatography; **GC**-**MS**—Gas-Chromatography-Mass Spectrometry; **LC-MS**—Liquid chromatography-mass spectrometry. Numerals indicate the kind of taro formulation and letters indicate the type of antioxidant compound or the method used for antioxidant evaluation.
